# Efficacy of Extended Time on Exams for College Students and Applicants With Attention-Deficit/Hyperactivity Disorder: Protocol for a Randomized, Counterbalanced Crossover and Within-Subject Trial

**DOI:** 10.2196/80271

**Published:** 2026-06-03

**Authors:** Lui Malcon, Alice Gomes Silva, Arthur Bezerra Falcão, Érica Bonganhi de Bem, Felipe Grevet, Flávia Wagner, Igor Duarte, Leonardo Barbedo, Maitê Schneider, Luis Augusto Rohde, Maurício Hoffmann, Arthur Caye

**Affiliations:** 1 Graduate Program in Psychiatry and Behavioral Health Universidade Federal do Rio Grande do Sul Porto Alegre, Rio Grande do Sul Brazil; 2 Department of Psychiary ADHD Outpatient Program & Developmental Psychiatry Program Hospital de Clínicas de Porto Alegre Porto Alegre, Rio Grande do Sul Brazil; 3 Department of Psychiatry Universidade de São Paulo São Paulo Brazil; 4 Instituto Nacional de Psiquiatria do Desenvolvimento para Crianças e Adolescentes São Paulo, São Paulo Brazil; 5 Medical Council Centro Universitário de Jaguariúna (UNIFAJ) & Centro Universitário Max Planck (UNIMAX) Jaguariuna, São Paulo Brazil; 6 Department of Neuropsychiatry Universidade Federal de Santa Maria Santa Maria, Rio Grande do Sul Brazil; 7 Care Policy and Evaluation Centre London School of Economics and Political Science London, England United Kingdom; 8 Center for Research and Innovation in Mental Health São Paulo, São Paulo Brazil

**Keywords:** attention-deficit/hyperactivity disorder, ADHD, college admission test, psychosocial intervention, special education, extended testing time, extra time

## Abstract

**Background:**

Extended time on academic exams is one of the most frequently granted accommodations for students with attention-deficit/hyperactivity disorder. However, there is a lack of empirical evidence supporting its effectiveness, particularly in the college population.

**Objective:**

This study aimed to assess the efficacy and specificity of extended test time as an accommodation for college and college-prospective students with attention-deficit/hyperactivity disorder.

**Methods:**

This study will use a randomized, counterbalanced crossover and within-subject experimental design, using convenience sampling to recruit participants. All participants will undergo clinical and neuropsychological assessments to confirm their diagnostic status and cognitive performance. Following these assessments, participants will be randomized to sequentially complete 3 versions of a test modeled on the Brazilian National High School Exam under standard time conditions, with 25% extra time and with 50% extra time.

**Results:**

The study was funded in February 2023. Data collection started in August 2023. As of March 2026, we have enrolled 103 participants. Data analysis was initiated in March 2026, and no projected timeline for the publication of results is currently available.

**Conclusions:**

The study will be the first of its kind outside the United States and one of the first to investigate the effectiveness of extended testing time at different dosages in the college population. Findings will provide insights into the impact of extended time accommodations in a distinct academic system, addressing gaps in previous research, such as different intervention doses and predictors of effectiveness.

**Trial Registration:**

ClinicalTrials.gov NCT06063382; https://clinicaltrials.gov/study/NCT06063382

**International Registered Report Identifier (IRRID):**

DERR1-10.2196/80271

## Introduction

Attention-deficit/hyperactivity disorder (ADHD) is a prevalent neurodevelopmental condition that affects approximately 5.29% of children and adolescents and 3.4% of adults worldwide [1]. Although symptoms persist into adulthood for a substantial proportion of individuals [2], most research remains disproportionately focused on the pediatric population.

Individuals with ADHD face academic challenges from childhood onward [3]. They tend to achieve lower educational levels [4-7] as compared to their unaffected peers, and those who enroll in higher education continue to exhibit higher dropout rates and lower academic performance [8,9]. In response to these challenges, clinical guidelines increasingly emphasize multidisciplinary approaches for the management of ADHD.

The legal framework surrounding disability rights has evolved significantly over the past decades. The Americans With Disabilities Act is a landmark civil rights law enacted in the United States in 1990 that addresses the rights of people with disabilities [10]. Less well-known legislations that opened the path to the Americans With Disabilities Act are the Rehabilitation Act of 1973 [11], the Individuals With Disabilities Education Act of 1975 [12], and the United Nations Declaration on the Rights of Disabled Persons in 1975 [13]. More recently, Brazil enacted the Law on the Inclusion of Persons With Disabilities in 2015 [14]. In accordance, a law was enacted in 2021 to ensure comprehensive support for students with learning disorders and ADHD [15]. These laws explicitly state the need for reasonable accommodations—environmental modifications that will guarantee persons with disabilities the exercise of rights on an equal basis to others without imposing a disproportionate burden [16]. In educational settings, a common accommodation is extended test-taking time, which has become widely implemented to level the playing field for students with ADHD in schools and universities.

Despite its widespread use, the empirical evidence supporting the efficacy and specificity of extended testing time remains limited. While existing findings suggest that extended time can benefit students under time pressure conditions, it is not exclusive to those with ADHD as it also helps students without academic difficulties [17]. This raises important questions about the validity and fairness of such accommodations as specialists argue that valid accommodations must produce significantly greater improvements for the target group compared to those not eligible for the accommodation [18,19].

A systematic review of educational accommodations for children and adolescents with ADHD found 9 studies evaluating the effectiveness of extended time [17], but they exhibited significant methodological limitations that warrant important consideration. First, only 2 of them had experimental designs [20,21]. Second, they were all carried out in the United States and relied on predominantly White samples. Third, most were conducted before 2007, the most recent one having been published 10 years ago [22]. When we look at studies focusing on the college population, we find that they are both scarce and inconsistent, varying in study design [23,24]. Furthermore, again, the existing research has been conducted exclusively in the United States, limiting the generalizability of findings to diverse educational systems and cultural contexts. These methodological gaps highlight the need for more robust research to determine whether extended testing time achieves its intended purpose. Considering the cognitive heterogeneity of individuals with ADHD [25], a variance in response to the accommodation within the clinical group is also possible—which has also not been addressed by previous studies. Another important aspect is determining how much extra time should be provided. Today, this decision is grounded in common sense reasoning.

Given these gaps, this study aims to assess the impact of additional test time on the performance of college and college-prospective students with and without ADHD. Using an experimental design, we will vary extended time conditions (25% and 50% extra time). We hypothesize that the performance of students of both groups will be superior when additional time is provided but that students with ADHD will potentially experience greater relative benefits. We anticipate that students’ performance will improve significantly more with 50% extra time. Furthermore, we hypothesize that students with ADHD who present greater cognitive and functional deficits will derive greater benefits from extended time accommodations.

## Methods

### Study Design

A randomized, counterbalanced crossover and within-subject superiority trial will be conducted to examine the relationship between test time (40 vs 50 vs 60 minutes) and group type (ADHD vs control) on test performance. The sample will include individuals aged 16 to 40 years who are first-year college students or preparing for college entrance exams. Inclusion and exclusion criteria are available in Textbox 1 [26,27]. Each participant will complete all test conditions in a counterbalanced order to control for sequence and learning effects, allowing for direct within-subject comparisons across time points and between diagnostic groups.

The primary outcome measure will be test performance, operationalized as the latent ability score derived from participants’ responses to the assessment items. The specific measurement variable will be the total number of correct responses, modeled using item response theory (IRT) to adjust for item difficulty, guessing behavior, and item discrimination parameters. The analysis metric will be the IRT‑estimated ability parameter (θ) representing each participant’s underlying proficiency level. The method of aggregation will involve calculating the mean and SD of the estimated ability scores across participants. The time point for this outcome will be immediately after completion of the test session.

**Inclusion and exclusion criteria.**

**Inclusion criteria**

- All participants must be aged 16 to 40 y and be first-year college students or enrolled in formal preparatory courses for entrance exams. Participants of the attention-deficit/hyperactivity disorder (ADHD) group must meet *Diagnostic and Statistical Manual of Mental Disorders, Fifth Edition, Text Revision (DSM-5-TR)*, criteria for ADHD confirmed by a licensed adult and child and adolescent psychiatrist.

**Exclusion criteria**

- Participants must not have an IQ below 80; auditory, visual, or neurological impairments that can interfere in the neuropsychological assessment; psychotic disorders; bipolar affective disorder; current and severe depressive episodes; risk of suicide; autism spectrum disorders; or learning disabilities.
- Additionally, participants of the control group must not present more than 3 symptoms of ADHD occurring frequently as described in the *DSM-5-TR* [26]. Although the diagnostic criteria require ≥5 symptoms in adults, recent studies report a fluctuating course of the disorder across its development [27].

IRT models incorporate 3 core components—the individual test taker, the characteristics of each test item, and the latent ability being measured—allowing for a more precise and psychometrically robust estimate of performance [28]. To compute the weighted score, we applied the *mirt* package (R Foundation for Statistical Computing) [29].

### Instruments

#### Overview

We will administer a comprehensive assessment protocol composed of a neuropsychological evaluation, a psychiatric evaluation, and a set of self‑report surveys. These instruments serve distinct analytic purposes, namely, eligibility screening, moderation analysis, and main outcome measurement. Moderation variables will not be used in the main results and are, instead, reserved for separate exploratory studies. Details of each instrument used in the study, including the instrument used in the intervention, are provided below.

#### Eligibility Screening Instruments

##### Sociodemographic Survey

This will be used to collect basic information, such as name, date of birth, sex and gender, socioeconomic status [30], address, contact information, nationality, place of birth, race (White, Black, Asian, “pardo,” or Indigenous), marital status, occupation and income (individual and household), school grade repetition, diagnosis of specific learning disabilities (LDs; dyslexia, dyscalculia, or dysgraphia), and diagnosis of autism spectrum disorder. An open-ended question was added to collect information about impairments.

##### Adult ADHD Self-Report Scale

The Adult ADHD Self-Report Scale (ASRS-18) [31] is a self-administered scale developed to assist in the diagnosis of ADHD in the adult population. It consists of 18 questions rated on a 5-point Likert scale (“never,” “rarely,” “sometimes,” “often,” and “very often”) divided into 2 parts: part A assesses inattention symptoms, whereas part B assesses hyperactivity and impulsivity symptoms. Responses marked as “often” or “very often” are considered clinically significant. The ASRS-18 has been adapted and translated for the Brazilian context [32].

##### Wechsler Abbreviated Scale of Intelligence 2-Subtest Format

The Wechsler Abbreviated Scale of Intelligence is an individually administered clinical instrument that assesses the intellectual ability of individuals aged 6 to 89 years. The short version, consisting of the vocabulary and matrix reasoning subtests, will be used to estimate the participants’ global IQ. The instrument has been translated and validated for the Brazilian context. It shows an excellent reliability coefficient (Spearman–Brown reliability coefficient, rₛᵦ=0.94) and internal consistency (Cronbach α=0.93). It also presents good test-retest reliability (*r*=0.81; corrected *r*=0.87) [33].

##### Structured Clinical Interview for the Diagnostic and Statistical Manual of Mental Disorders, Fifth Edition

The Structured Clinical Interview for the *Diagnostic and Statistical Manual of Mental Disorders, Fifth Edition* (*DSM-5*) [34], is a semistructured interview guide for making the major *DSM-5* diagnoses. It is administered by a clinician or trained mental health professional who is familiar with the *DSM-5* classification and diagnostic criteria.

#### Moderating Variable Instruments

##### Word and Pseudoword Reading Task

The word and pseudoword reading task [35] will be used to screen for dyslexia. This instrument is based on cognitive neuropsychology information processing models and consists of 24 regular words, 24 irregular words, and 24 pseudowords. Construct validity was evaluated by correlating the total score and subcomponent scores (regular words, irregular words, long words, short words, frequent words, nonfrequent words, short pseudowords, and long pseudowords) with different age groups, years of formal education, and study habits. Results in the adult population were statistically significant for schooling (*P*≤.05; r=0.22-0.6) and study habits (*P*≤.05; r=0.24-0.34). Convergent validity was evaluated by correlating the total, words and pseudowords scores with similar instruments (Brief Neuropsychological Assessment Battery for patients with expressive aphasia and the token test). Results were significant for nearly all correlations with the token test (*P*≤.01; r=0.41-0.77) and for several Brief Neuropsychological Assessment Battery for patients with expressive aphasia tasks (*P*≤.05; r=0.41-0.61). The instrument showed excellent internal consistency (Cronbach α=0.929), and all subscales correlated with each other (*P*≤.01; r=0.327-0.954). Furthermore, it presented high interclass correlation between evaluators for all but 1 variable (*P*≤.01; r=0.882-0.98) [36].

##### Liebowitz Social Anxiety Scale

The Liebowitz Social Anxiety Scale (LSAS) [37] is a self-administered scale developed to aid in the diagnosis of social anxiety disorder. The LSAS consists of 24 items rated from 0 to 3 for both fear or anxiety and avoidance. The LSAS has been validated for the Brazilian context, with very good internal consistency for both the total score (Cronbach α=0.96) and subscales (fear subscale: Cronbach α=0.94; avoidance scale: Cronbach α=0.93) [38]. It also shows excellent discrimination between case and noncase groups, with area under the curve values greater than 0.90, sensitivity greater than 0.91, and specificity greater than 0.79. Similarly, the discrimination between noncases and subclinical groups remains high (area under the curve=0.86; sensitivity>0.87; specificity>0.76) [39].

The following items were selected, along with their avoidance counterparts, as they are theoretically relevant to academic test performance: 8—fear or anxiety when working and being observed, 9—fear or anxiety when writing and being observed, and 19—fear or anxiety when taking a test.

##### Barkley Deficits in Executive Functioning Scale

The Barkley Deficits in Executive Functioning Scale (BDEFS) [40] is a gold-standard self-report scale designed to assess executive function deficits in the daily activities of adults. The functions evaluated are time management, organization and problem-solving, self-control, self-motivation, and emotional self-regulation. The scale has been translated, adapted, and validated for the Brazilian context [41], with a Cronbach α of 0.961 for all scales and from 0.790 to 0.970 for subscales, indicating satisfactory internal consistency. The mean of the differences between the total scores on the BDEFS Brazilian Portuguese version and the total scores on the BDEFS English version was −0.16 (mean −0.16; SD=9.651), and the maximum differences were −4.1437 and 3.8237, indicating high clinical agreement between versions. Spearman correlations among the BDEFS Brazilian Portuguese version, Barratt Impulsiveness Scale, and ASRS-18 were all statistically significant (*P*<.05).

##### Weiss Functional Impairment Rating Scale–Self-Report

The Weiss Functional Impairment Rating Scale–Self-Report [42] collects respondents’ perspectives on their own functioning across 7 domains: family (8 items), work (11 items), academic (10 items), daily life skills (12 items), self-concept (5 items), social life (9 items), and risk (14 items). Respondents rate each item based on the level of functional impact in the past month on a 4-point Likert scale, with an additional “not applicable” option. The adult version is not yet available in Portuguese; however, translation has been authorized for use in this study.

The original scale presented a Cronbach α range between 0.85 and 0.96, evidencing good to excellent internal consistency. It also showed excellent concurrent validity, with all correlations significant (*P*<.001) and large effect sizes (*r*>0.50) [43].

##### Conners' Continuous Performance Test

The Conners' Continuous Performance Test [44] is used to assess sustained attention, processing speed, reaction time, and impulsivity. During this test, participants are presented with a series of visuals on a computer screen and are instructed to press a button when they see specific stimuli while inhibiting responses to other stimuli. A total of 360 letters appear on the screen one at a time, each displayed for approximately 250 ms. These letters are presented in 18 consecutive blocks of 20 trials, with interblock intervals randomized to 1, 2, or 4 seconds. Participants must press a button each time a letter appears except for the letter X. Non-X letters appear 90% of the time. Key indexes include hits, which measure the number of appropriate responses to target stimuli; omissions, indicating failures to respond to a target letter; and commissions, which track responses to nontarget stimuli. Other notable indexes are detectability, assessing the ability to discriminate between targets and nontargets, and response style, which evaluates the balance between speed and accuracy in responses. Additionally, the test generates indexes of variability, perseverations, and various block and interstimulus interval changes, which provide insights into response consistency, impulsivity, and vigilance.

A systematic review found a moderate 3-month test-retest reliability (0.55-0.84); mixed sensitivity, with findings varying from 33.3% to 71%; and mixed specificity, varying from 51.7% to 91.7%. Discriminant validity was evaluated in samples with comorbid obsessive-compulsive disorder (*R*^2^=0.16) and depression (β=−0.226) [45].

##### COMTEXT-Uni: Textual Reading Comprehension Assessment for College Students Scale

Developed as part of the research project entitled “Reading Comprehension: Assessment and Intervention” (Universidade Federal do Rio Grande do Sul, School of Education, coordinated by Dr Helena Vellinho Corso), the reading comprehension assessment instrument COMTEXT-Uni is undergoing standardization. It is based on cognitive neuropsychology models and assesses university-level adolescents and young adults with typical development, suspected reading comprehension difficulties, and other conditions that may affect reading processes. It consists of a narrative text followed by 3 tasks: oral retelling, multiple-choice questionnaire (with 5 literal and 5 inferential questions), and oral reading.

##### Digit Span and Coding Subtests of the Wechsler Adult Intelligence Scale

The Wechsler Adult Intelligence Scale is an individually administered clinical instrument that assesses the intellectual ability of individuals aged 16 to 89 years. The digit span subtest will be used to measure auditory working memory, and the coding subtest will be used as a measure of processing speed. The Wechsler Adult Intelligence Scale has been translated and validated for the Brazilian context. The digit span subtest shows moderate reliability (*r*=0.61; corrected *r*=0.66) and good internal consistency (Cronbach α=0.81). The coding subtest presents a higher test-retest reliability (*r*=0.70) and satisfactory internal consistency (Cronbach α=0.70) [46].

#### Primary Outcome Measure Instrument

The primary outcome measure will be the weighted test scores of a standardized task specifically constructed for this study. The weighted test scores will be calculated using the 3-parameter IRT. IRT is a statistical modeling approach that links individuals’ ability levels to their likelihood of responding correctly to each item, incorporating parameters for item difficulty, discrimination, and guessing. The guessing parameter measures the likelihood of a student guessing the correct answer purely by chance based on number of answer choices, item design, test taker behavior, and empirical calibration from response data; the discrimination parameter measures how well a question differentiates between high-performing and low-performing students by calculating the correlation between a student’s performance on a specific question and their overall test performance; and the difficulty parameter measures how hard or easy a particular question is based on the percentage of correct responses from all students [47].

The standardized task was designed based on the Brazilian National High School Exam (ENEM), which was constructed using the 3-parameter IRT. In this study, we selected a guessing parameter below 0.2, a discrimination parameter above 2, and a 2:1 ratio (difficult to easy) difficulty parameter. These indexes allow us not only to construct an exam equivalent to the real-world scenario but also to have 3 different tests with the same difficulty level. Each version consists of 12 questions covering different areas of knowledge using identical items that appear in the original ENEM (see Multimedia Appendix 1).

We conducted a pilot exam with 30 students to verify whether the 3 versions of the task were equivalent. First, we examined normality regarding exam scores using the Shapiro-Wilk test. As we found a signal for a nonnormal distribution, we used a Friedman ANOVA to assess group differences and paired Wilcoxon signed-rank tests to compare the scores between each pair of exams (A vs B, A vs C, and B vs C). In addition, we used a Shapiro-Wilk test to assess the normality of the scores for each administration order. As the distribution was normal, we conducted a repeated-measure ANOVA to examine whether the order of administration affected the scores. We inspected paired differences between orders using paired 2-tailed *t* tests.

### Psychiatric Evaluation

To confirm the ADHD diagnosis and control for comorbidities, trained psychiatrists will conduct an online clinical interview using selected sections of the Structured Clinical Interview for the *DSM-5*, namely, ADHD, depressive episodes (current and past), manic episodes (current and past), and psychotic symptoms.

### Neuropsychological Assessment

A neuropsychological assessment will be administered by trained professionals using the following instruments: the Conners' Continuous Performance Test–Second Edition, COMTEXT, the Wechsler Abbreviated Scale of Intelligence digit span task and coding subtests, and the word and pseudoword reading task. The tests will be administered in a fixed sequence for all participants.

### Procedures

#### Overview

Data will be collected and managed using REDCap (Research Electronic Data Capture; Vanderbilt University), a secure, web-based software designed to support data capture for research studies [48]. The intervention will be conducted at Universidade Federal do Rio Grande do Sul, the neuropsychological assessments will be conducted at Hospital de Clínicas de Porto Alegre’s Clinical Research Center, and the psychiatric assessments will take place online using a secure web-based videoconferencing platform. To enhance recruitment efficiency, we will adopt 2 different strategies for students with ADHD and controls.

#### Data Collection for Participants With ADHD

Initially, participants with potential ADHD will be recruited via public call using convenience sampling. Announcements will be shared through the researchers’ and partner institutions’ (universities and preparatory schools for college entrance exams in the city of Porto Alegre, capital of the southernmost state of Brazil) social media channels.

Interested individuals will contact the staff through social media platforms, email, or other instant messaging apps and will receive a REDCap survey link containing an informed consent form and the following instruments: sociodemographic survey, LSAS, BDEFS, Weiss Functional Impairment Rating Scale–Self-Report, and ASRS-18. Following the screening, participants who report 5 or more symptoms in the ASRS-18 will undergo psychiatric and neuropsychological assessments. Those who meet the inclusion criteria for the ADHD group will take the standardized exam.

#### Data Collection for Controls

Due to logistical constraints related to team resources, the sequence of procedures for the control group will differ from that used for the ADHD group. We will partner with colleges and preparatory schools to present the research protocol during regular classes. Interested individuals who report no prior ADHD diagnosis will be invited to participate in the on-site intervention on a predetermined date. Sessions will be held in the exact same manner as done with participants with ADHD. Immediately before the test session, potential control group students will complete a printed version of the ASRS-18. On the basis of these results, students exhibiting less than 4 symptoms on the scale will be invited to participate in the subsequent stages of the study (REDCap surveys, psychiatric assessment, and neuropsychological testing).

#### Intervention Procedures

To minimize fatigue, the intervention will be administered on a separate day from the psychiatric and neuropsychological assessments in groups of 5 to 15 participants and will always be administered in the morning. To assess participants’ optimal performance, they will be instructed to take medications as usual. Information regarding dose and time of intake will be collected on the day of the intervention. Each session will be facilitated by at least one assistant and the principal investigator (PI). Sessions will be conducted in quiet university or preparatory school classrooms to maintain a naturalistic environment.

**Figure 1:**
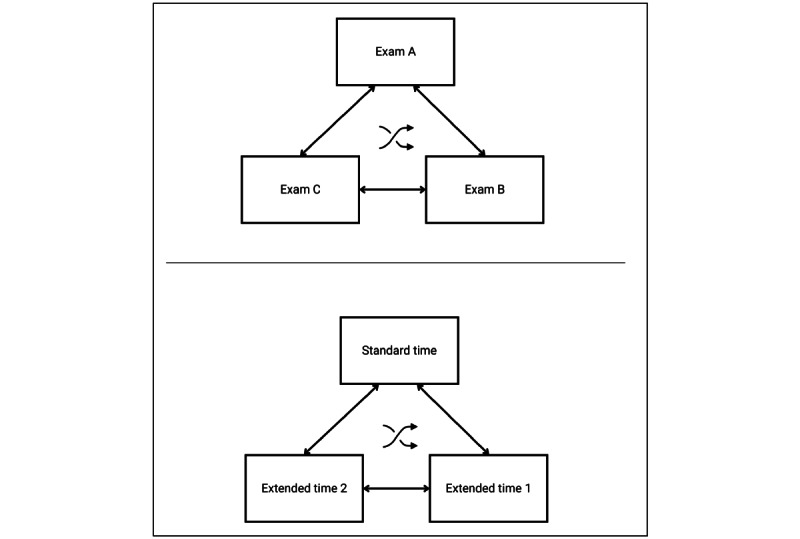
Sequence of the exam versions and time conditions randomized for each participant.

Each participant will complete an academic exam with 3 different versions (A, B, and C) of similar difficulty and length. Each version will be paired with 1 of 3 distinct time conditions: 40 minutes (standard), 50 minutes (25% extra time), and 60 minutes (50% extra time). To control for potential confounding variables and ensure that the results are not biased by the order of conditions or exam versions, both the sequence of the exam versions and the time conditions will be randomized for each participant, as illustrated in Figure 1. This counterbalanced design ensures that each participant experiences all 3 time conditions (40, 50, and 60 minutes) and each exam version (A, B, and C) in a different order, preventing any systematic influence from order effects on the outcomes. Sequences of task orders will be computer generated using a random number generator and then counterbalanced so that each condition appears equally often in each position across participants.

At the beginning of each session, the PI will explain the procedure to the participants. The designated time limits will be informed on the first page of the exams. When the time limit of the exam is reached, participants will be stopped and handed the next exam. Only answers marked on a separate answer sheet will be considered. The time will be tracked on a whiteboard. Participants will be instructed to remain until the end of each exam regardless of finishing before the allocated time to minimize the risk of rushing through the test. Participants will start each exam at the designated beginning time.

### Data Analysis

Analyses will be conducted using SPSS Statistics (IBM Corp) [49]. We will first characterize the sample by group (ADHD vs control), reporting means and SDs for continuous variables and counts and percentages for categorical variables. Between‑group differences in baseline characteristics will be tested using *t* tests (Welch *t* test when variances are unequal) for continuous measures (eg, age and IQ) and chi-square tests (or Fisher exact tests when indicated) for categorical variables (sex, educational level, race, and socioeconomic status). Statistical significance for both data analysis and statistical analysis will be set at a *P* value of .05 or less.

### Sample Size Calculation

For a small to moderate effect size (*d*=0.45), 80 participants are needed to achieve 80% power at a *P* value of .05 or less for within-subject analyses. Participants will be equally divided into 2 groups: clinical group (with ADHD) and control group (without ADHD), with 40 participants in each group.

### Statistical Analysis

Primary performance analyses will use standardized, IRT‑equated scores from the ENEM‑based exams; raw counts will not be analyzed. Estimated marginal means with 95% CIs will summarize group profiles and within‑person gains. We will also conduct a sensitivity analysis excluding medicated individuals.

Group comparisons at each time limit (40, 50, and 60 minutes) will be performed using Welch *t* tests with Cohen *d* and 95% CIs. Within‑group time effects will be evaluated via repeated‑measure ANOVA with time (40, 50, and 60 minutes) as a within‑subject factor; the Mauchly test will assess sphericity, and Greenhouse-Geisser corrections will be applied when necessary. Planned paired contrasts (50 vs 40 and 60 vs 40 minutes) will use Bonferroni adjustment, and partial η^2^ will index omnibus within‑subject effects.

To directly test whether extended time benefits participants with ADHD more than controls, we will fit a linear mixed‑effects model with a random intercept for participant, coding time continuously in minutes (40, 50, and 60) so slopes are interpretable per minute. Fixed effects will use Satterthwaite df, and interaction tests will be corroborated with likelihood ratio comparisons under maximum likelihood.

Assumptions will be examined using Shapiro-Wilk tests and *Q*-*Q* plots (normality), Levene tests (homogeneity), and visual inspection of mixed‑model residuals. When ANOVA assumptions are not met, inference will prioritize mixed‑model results. Analyses will be conducted in R (*lme4* and *lmerTest*, *emmeans*, and *afex* packages; R Foundation for Statistical Computing).

Missing data will be handled using multiple imputation under the assumption that data are missing at random, and all analyses will be conducted on the imputed datasets using an intention‑to‑treat approach.

### Ethical Considerations

Approved by the Hospital de Clínicas de Porto Alegre ethics committee, the study ensures confidentiality, informed consent, and compliance with National Health Council Resolutions 466/2012 and 510/2016, as well as the General Data Protection Law (Law 13,709/2018). Participants may request feedback, and those with clinical needs will be referred to specialized services. Access to task order sequence information will be restricted to the PI, who will generate the allocation sequence through a secure web‑based system before the intervention. The resulting files will be stored on a password‑protected computer accessible only to the PI. The files will become accessible to the study team only at the time of the intervention, ensuring that allocation information is revealed strictly when operationally necessary. To motivate optimal performance and simulate real-world competition, bookstore gift cards will be awarded to the top 3 scorers in each area of entrance exams, namely, health sciences, such as medicine, nursing, and biomedicine; engineering and technologies, such as civil engineering and software engineering; humanities and social sciences, such as law, psychology, and pedagogy; exact and natural sciences, including mathematics, physics, chemistry, and biology; and arts and communication, such as design, journalism, and advertising. This incentive, unrelated to participation recruitment, will only be revealed at the beginning of the intervention session.

## Results

### Overview

The study was funded in February 2023, and data collection started in August 2023. Data analysis began in March 2026, and no projected timeline for the publication of results is currently available. As of March 2026, a total of 103 participants have been enrolled and allocated to either the control group (n=43, 41.7%) or the ADHD group (n=60, 58.3%) based on diagnosis.

### Participant Flow

**Figure 2:**
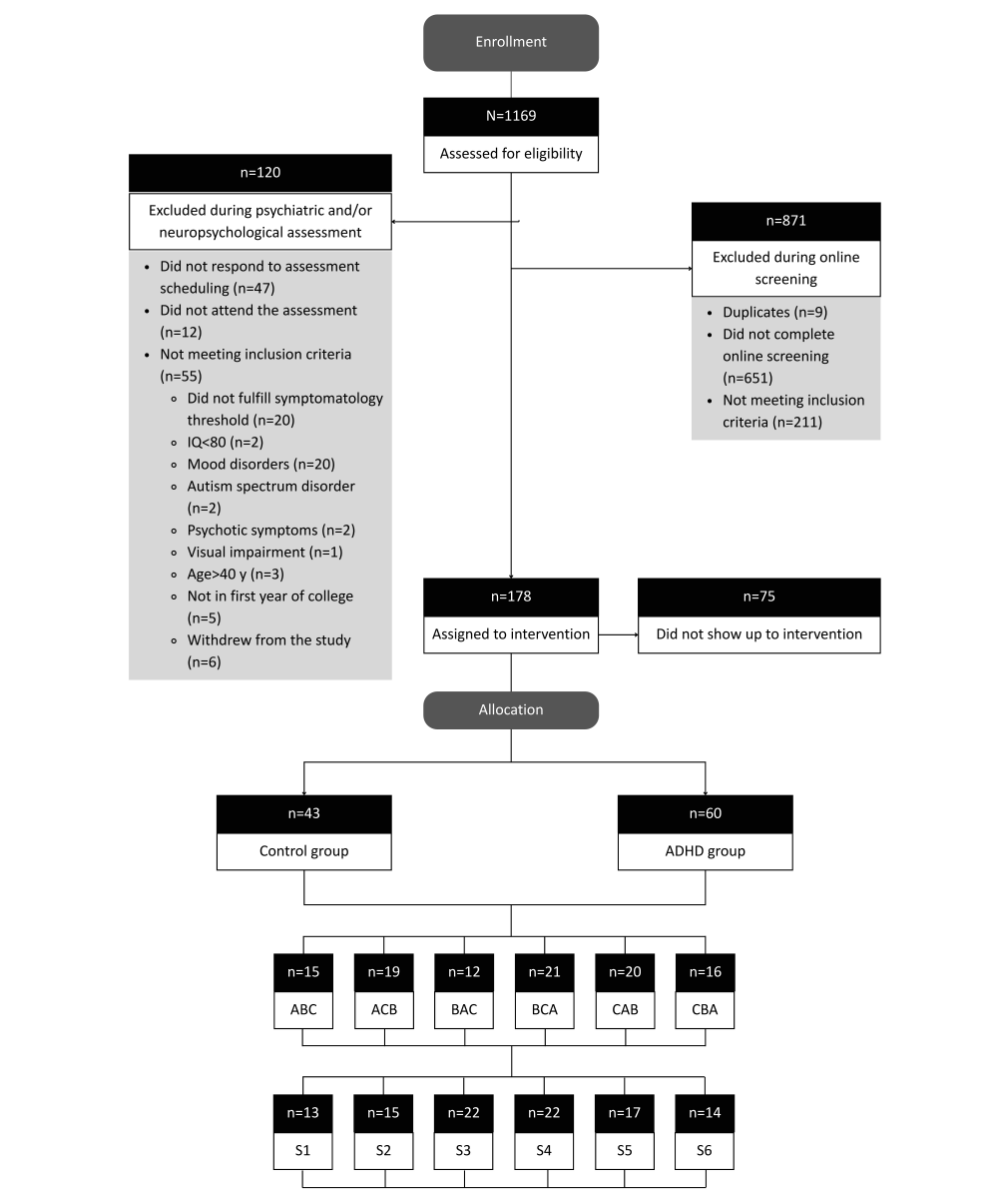
Participant flow. Allocation was randomized across exam order (6 possible sequences; eg, ABC, ACB, BAC, BCA, CAB, and CBA) and time allocation order (6 possible sequences; sequence 1 [S1]=40-50-60; sequence 2 [S2]=40-60-50; sequence 3 [S3]=50-40-60; sequence 4 [S4]=50-60-40; sequence 5 [S5]=60-40-50; sequence 6 [S6]=60-50-40). The final distribution included both the attention-deficit/hyperactivity disorder (ADHD) and control groups.

A total of 1169 individuals were initially assessed for eligibility. Of these 1169 individuals, 871 (74.5%) were excluded during the online screening phase due to duplicate entries (n=9, 1%), failure to complete the screening questionnaire (n=651, 74.7%), or not meeting the inclusion criteria (n=211, 24.2%). The remaining 298 participants were invited for a psychiatric and neuropsychological assessment, after which an additional 120 (40.3%) individuals were excluded. Reasons for exclusion at this stage included failure to respond to scheduling attempts (47/120, 39.2%) or not attending the assessment (12/120, 10%), as well as not meeting the clinical or demographic inclusion criteria (55/120, 45.8%), such as having insufficient symptom severity (20/55, 36.4%), mood disorders (20/55, 36.4%), autism spectrum disorder (2/55, 3.6%), psychotic symptoms (2/55, 3.6%), or visual impairment (1/55, 1.8%); being older than 40 years (3/55, 5.5%); or having an educational level above the eligible range (5/55, 9.1%) or an IQ below 80 (2/55, 3.6%). IQ was assessed through a neuropsychological assessment as described previously. Additionally, 5% (6/120) of the participants at this stage withdrew from the study. A total of 178 participants were subsequently assigned to the intervention; however, 75 (42.1%) did not attend the intervention sessions (Figure 2).

### Pilot Study

No significant differences were found among the 3 task versions or across administration orders (Table 1).

**Table 1:** Test type and order comparisons.

Comparison		Statistical test		Statistic		*P* value	Adjusted *P* value
**Test type**							
	A vs B	Paired Wilcoxon	*V*=145		.07		.22
	A vs C	Paired Wilcoxon	*V*=201		.53		>.99
	B vs C	Paired Wilcoxon	*V*=310		.11		.34
	A, B, and C (global)	Friedman	*χ*^2^_2_=3.9		.15		—^a^
**Order^b^**							
	1 vs 2	Paired *t* test	t_29_=0.22		.83		>.99
	1 vs 3	Paired *t* test	t_29_=−0.19		.85		>.99
	2 vs 3	Paired *t* test	t_29_=−0.40		.70		>.99
	1, 2, and 3 (global)	RM^c^ ANOVA	*F*_2,58_=0.11		.90		—

^a^Adjusted *P* values are not reported for the omnibus test, as this procedure entails only a single global comparison and therefore does not require correction for multiple testing.

^b^Order indicates the position in which each test was administered (eg, 1=test delivered first). Because test type and test order were randomized independently, any test (A, B, or C) could appear in any order position.

^c^RM: repeated measures.

## Discussion

### Expected Findings

This trial protocol describes a controlled experimental study designed to evaluate whether extended testing time confers a differential benefit to students with ADHD compared to students without ADHD. We hypothesize that extended time will improve overall test performance and that the magnitude of improvement will be superior for the ADHD group. We anticipate that students’ performance will improve significantly more with 50% extra time. Furthermore, we hypothesize that students with ADHD who present greater cognitive and functional deficits will derive greater benefits from extended time accommodations.

In this paper, we propose a study with an experimental design and the development of a standardized task that can be replicated to investigate this issue in college students. The protocol describes a much more robust methodology than those used in prior studies. Lovett and Leja [24] did not include participants with ADHD in their sample. Rather, they examined the association between the effects of extended time and the severity of ADHD symptoms in a nonclinical sample. In addition, the study used only a very short reading comprehension task of 10 minutes, with 5 additional minutes to complete the assignment. Neuropsychological tests were also administered immediately before the session, which may have influenced the participants’ performance. We will administer the neuropsychological assessment on a different day to avoid possible fatigue or carryover effects that could confound test outcomes. Miller et al [23] adopted a similar task to that used by Lovett and Leja [24], also with a short duration (20 minutes). The task developed for our study has a standard time of 40 minutes, with 25% and 50% extra time implemented across 3 parallel versions of the task. In contrast, the previous studies applied the time extension to a single version of the task. This was only possible because we designed the task using the IRT, which allowed us to create psychometrically equivalent parallel versions. Furthermore, the weighted score produced by the IRT provides a more precise estimate of the participants’ abilities. Finally, to the best of our knowledge, our study will be the first of its kind to be conducted in Latin America, which can inform about the effectiveness of extended time in a diverse academic system.

Despite its contributions, our study has limitations. Because our sample is limited to a specific region, it may not be representative of the entire country, thereby restricting the generalizability of our findings. In addition, we excluded participants with LDs. As these are conditions more common among students with ADHD and they possibly benefit from extended testing time, their presence might result in a cofounding variable difficult to adjust for in analyses considering sample size and other included confounders. However, excluding these individuals diminishes the ecological validity of our study. We encourage further studies to examine broader samples, including students with both ADHD and LDs, to determine independent and interactive effects. Future research should also explore whether students continue to require extra time while on pharmacotherapy and assess accommodations across diverse educational systems to help refine policies and ensure that accommodations are both equitable and effective, addressing the diverse needs of students with disabilities.

Finally, most of our sample comes from high-ranking universities or preparatory schools, which may skew academic performance higher than that of the general population and, thus, reduce the need for extra testing time. To mitigate this limitation, we have incorporated a comprehensive neuropsychological assessment to identify potential biases; the implications of these findings will be discussed. Nonetheless, it is important to note that many institutions grant testing accommodations based solely on a medical certificate without requiring documentation of IQ or academic performance. This includes the National Institute for Educational Studies, which administers the Brazilian college‑entry examination (ENEM). Such practices underscores the importance of the research, which aims to provide valuable insights into the factors that may influence the need for accommodations for people with ADHD, supporting the development of more inclusive and equitable testing practices.

Plans to disseminate this study’s findings include meetings with academics and faculty members from the participating universities, dissemination through regional and national media outlets, and presentation of the results at scientific events.

### Data Monitoring and Oversight

Given the minimal risk and noninvasive nature of the intervention and the small, single-center design, a data monitoring committee is not warranted. No formal interim monitoring of trial conduct is planned. Oversight of study procedures, data integrity, and protocol adherence will be performed continuously by the PI, and the study will remain under the supervision of the institutional ethics committee.

## Appendix

Multimedia Appendix 1 Translated exam booklets A, B, and C used in the intervention.

## Abbreviations

ADHD: attention-deficit/hyperactivity disorder
ASRS-18: Adult Attention-Deficit/Hyperactivity Disorder Self-Report Scale
BDEFS: Barkley Deficits in Executive Functioning Scale
DSM-5: Diagnostic and Statistical Manual of Mental Disorders, Fifth Edition
ENEM: National High School Exam
IRT: item response theory
LD: learning disability
LSAS: Liebowitz Social Anxiety Scale
PI: principal investigator
REDCap: Research Electronic Data Capture
