# Supplementary material for: Efficacy of Extended Time on Exams for College Students and Applicants With Attention-Deficit/Hyperactivity Disorder: Protocol for a Randomized, Counterbalanced Crossover and Within-Subject Trial
Source: JMIR Res Protoc. 2026 Jun 3;15:e80271. doi: 10.2196/80271 (PMC13276468; doi:10.2196/80271)
Supplement: Multimedia Appendix 1 [file resprot_v15i1e80271_app1.docx]

# **Supplementary Files**

1. Exam Booklets
   1.1) Exam A

Full name: _______________________________________ Date: _______________

Course: __________________________

Did you take any medication today? If so, specify: ________________________

Time the medication was taken:_______________ Dosage:_____________________

**PLEASE READ THE FOLLOWING INSTRUCTIONS CAREFULLY:**

1. This QUESTION BOOKLET contains 12 questions from various areas of knowledge.
2. Each question has 5 options, but only one is correct.
3. The total time available for the test is ______________.
4. Allocate enough time to fill out the ANSWER SHEET.
5. Drafts and markings in the QUESTION BOOKLET will not be considered for evaluation.
6. Once you finish the test, signal the proctor and hand in both the QUESTION BOOKLET and the ANSWER SHEET.

**Question 1**

Two major historical events made a case like Menocchio’s possible: the invention of the printing press and the Reformation. The printing press allowed him to confront books with the oral tradition in which he was raised and provided him with the words to organize the jumble of ideas and fantasies coexisting within him. The Reformation gave him the audacity to share his thoughts with the village priest, fellow villagers, and inquisitors—even though he was unable to express everything before the pope, cardinals, and princes, as he wished.

GINZBURG, C. The Cheese and the Worms: The Cosmos of a Sixteenth-Century Miller. São Paulo: Companhia das Letras, 2006.

The historical events mentioned helped this individual in the 16th century to rethink the Catholic worldview by enabling:

1. Public access to royal libraries.
2. The Baroque sophistication of liturgical rituals.
3. The popular acceptance of secular education.
4. The autonomous interpretation of biblical texts.
5. The doctrinal correction of medieval heresies.

**Question 2**

**Salamanca Declaration – 1994**
We believe and proclaim that: every child has a fundamental right to education and should be given the opportunity to reach and maintain an appropriate level of learning; every child has unique characteristics, interests, abilities, and learning needs; educational systems should be designed and educational programs implemented to consider the vast diversity of such characteristics and needs.

Available at: <http://portal.mec.gov.br>. Accessed on: Oct. 4, 2015.

As a signatory of the cited Declaration, Brazil has committed to developing educational public policies that promote:

1. The creation of privileges.
2. Cost containment.
3. The plurality of individuals.
4. Curriculum standardization.
5. The valorization of meritocracy.

**Question 3**

In recent decades, there has been a significant feminization of the workforce. While male participation has seen little growth since the 1970s, the increased presence of women has been a defining feature. However, this female presence is concentrated in precarious employment sectors, where exploitation is often more pronounced.

NOGUEIRA, C. M. The Telemarketing Workers: A New Gender Division of Labor? In: ANTUNES, R. et al. Infoproletarians: The Real Degradation of Virtual Work. São Paulo: Boitempo, 2009.

The transformation described in the text has been insufficient to establish equal opportunity due to:

1. The stagnation of acquired rights and anachronism of the present legislation.
2. The maintenance of managerial status quo and family socialization patterns.
3. The dismantling of patriarchal heritage and shifts in occupational profiles.
4. Conflicts in union composition and political-party representation.
5. The demands for professional development and managerial competence.

**Question 4**

Green Chemistry is a branch of chemistry that promotes the development of efficient processes that convert most of the reagents into products in a faster and more selective way, using fewer reagents, producing only the desired product while avoiding byproducts, and utilizing environmentally friendly solvents. Thus, industries could avoid problems related to environmental pollution and waste of water and energy.

A process that follows all the principles of Green Chemistry is represented by:

1. A + B + C → D (reaction occurs at high pressure).
2. A + B → C + D (reaction is strongly endothermic).
3. A + 3B → C (reaction occurs using an organic solvent).
4. 3A + 2B → 2C → 3D + 2E (reaction occurs at atmospheric pressure).
5. A + 1/2B → C (reaction occurs using a catalyst containing a non-toxic metal).

**Question 5**

To move and obtain food, some mammals, such as bats and dolphins, rely on a sophisticated biological ability to detect the position of objects and animals through the emission and reception of ultrasonic waves.

The wave phenomenon that enables this biological capability is:

1. Reflection.
2. Diffraction.
3. Refraction.
4. Dispersion.
5. Polarization.

**Question 6**

A person notices that the battery of their vehicle becomes discharged after five days without use. At the beginning of this period, the battery was functioning normally and had its full nominal charge of 60 Ah. Considering the possibility of a leakage current, which persists even when the vehicle’s electrical devices are turned off, they connect a digital ammeter to the vehicle’s circuit.

Which of the diagrams correctly indicates how the ammeter should be connected and the reading it should display?


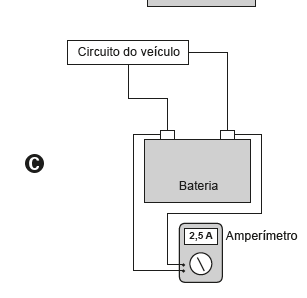

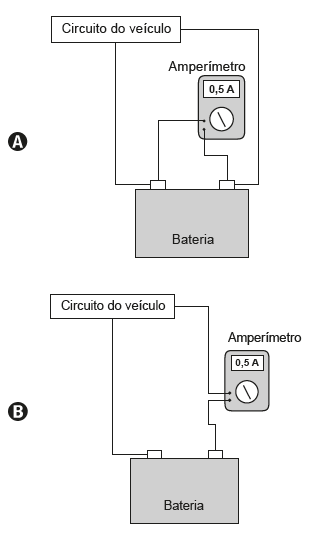

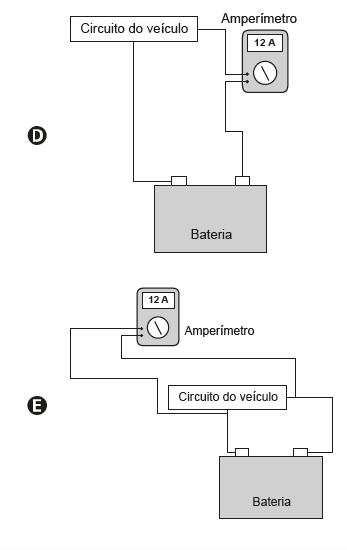


**Question 7**

**The Gold of the 21st Century**

Cerium, gadolinium, lutetium, promethium, and erbium; samarium, terbium, and dysprosium; holmium, thulium, and ytterbium. This list of strange and little-known names might seem like a soccer team lineup, which would also have lanthanum, neodymium, praseodymium, europium, scandium, and yttrium on the bench. However, these 17 metals, known as rare earth elements, are part of the daily lives of almost all humans on the planet. Called by many “the gold of the 21st century,” “elements of the future,” or “vitamins of industry,” they are found in materials used to manufacture light bulbs, computer screens, tablets, cell phones, electric car motors, batteries, and even wind turbines. Despite their numerous applications, Brazil, which has the world's second-largest reserves of these metals, ceased their extraction and use in 2002. Now, the country is reconsidering resuming their exploitation.

SILVEIRA, E. Available at: www.revistaplaneta.com.br. Accessed on: Dec. 6, 2017 (adapted).

The quotation marks in the text indicate metaphorical expressions intentionally used by the author to:

1. Convey an ironic tone in the report.
2. Incorporate expert citations into the report.
3. Assign greater value to the metals discussed in the report.
4. Clarify scientific terms used in the report.
5. Indicate the adoption of terminology from another scientific field.

**Question 8**

"I’m going away to Pasárgada" was the poem with the longest gestation in my entire work. I first saw the name Pasárgada when I was sixteen, in a Greek author. [...] This name, which means 'field of the Persians' or 'treasure of the Persians,' sparked in my imagination a fabulous landscape, a land of delights, similar to the one in Baudelaire’s L’invitation au voyage. More than twenty years later, when I lived alone in my house on Rua do Curvelo, in a moment of deep discouragement and the most acute feeling of everything I had not done in my life due to illness, this absurd outcry suddenly emerged from my subconscious: 'I’m going away to Pasárgada!' I felt in that phrase the first cell of a poem and tried to write it, but I failed. A few years later, under similar circumstances of despondency and boredom, the same expression of escape from the 'dull life' occurred to me. This time, the poem came effortlessly, as if it were already fully formed inside me. I like this poem because, in essence, it represents my entire life; [...] I am not an architect, as my father wished, I did not build any houses, but I reconstructed—not in an imperfect form in this world of appearances—but rather a noble city, which is no longer Cyrus’s Pasárgada, but my Pasárgada."

BANDEIRA, M. Itinerary of Pasárgada. Rio de Janeiro: Nova Fronteira; Brasília: INL, 1984.

The communicative processes involve the active presence of multiple elements of communication, among which the functions of language stand out. In this fragment, the predominant language function is:

1. Emotive, because the poet expresses the anguish that led him to poetic creation.
2. Referential, because the text provides information about the origin of the name used in a famous poem by Bandeira.
3. Metalinguistic, because the poet comments on the genesis and writing process of one of his poems.
4. Poetic, because the text discusses the aesthetic elements of one of Bandeira’s most famous poems.
5. Conative, because the poet tries to convince readers of his difficulty in composing a poem.

**Question 9**

"I own companies and am entitled to a visa to go to New York. The money that rains in New York is for people with purchasing power. People who have a visa from the American consulate. The money that rains in New York is also for New Yorkers. It’s thousands of dollars. [...] I am going to New York, where it is raining money. I am a great administrator. Yes, it is raining money in New York. They said so on the radio. I see pedestrians invading the lane where my red German-imported car is driving. I see domestic cars driving in the lane where my red German-imported car is driving. When I arrive in New York, I will take action."

SANT’ANNA, A. The Imported Red Car of Noah. In: MORICONI, I. (Org.). The Hundred Best Short Stories. Rio de Janeiro: Objetiva, 2001.

The repetition and short sentences are important linguistic procedures for understanding the text's theme, as they:

1. Express the futility of the narrator’s discourse on power and status.
2. Conceal the lack of depth in the existential anxieties narrated.
3. Ironize Brazilians' admiration of North American culture.
4. Make explicit the financial greed of contemporary capitalism.
5. Criticize the social stereotypes of elitist worldviews.

**Question 10**

A cosmetics store manager put five different types of perfume up for sale, maintaining equal stock quantities for each type. The inventory control department provided the manager with graphical records detailing the unit prices of each perfume (in Brazilian reais) and the percentage sold in November.


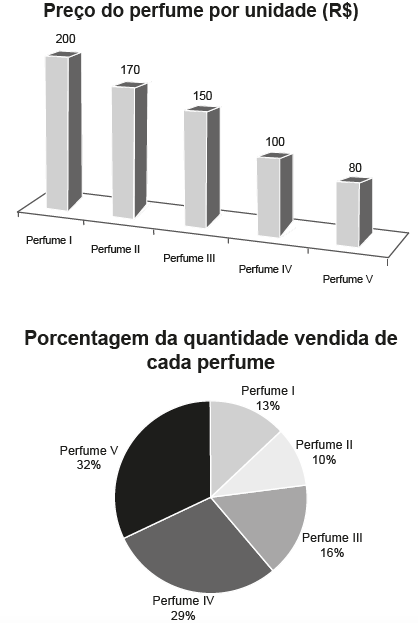


With the arrival of the end of the year and increased sales, management intends to restock the type of perfume that generated the highest total revenue (in reais) in November.

Under these conditions, which type of perfume should have the highest restocking priority?

1. I
2. II
3. III
4. IV
5. V

**Question 11**

A faucet is dripping water into a bucket with an 18-liter capacity. At the current moment, the bucket is filled to 50% of its capacity. Every second, 5 drops of water fall from the faucet, and each drop contains, on average, 5 × 10⁻² mL of water.

How much time, in hours, will it take to completely fill the bucket from its current level?

1. 2 × 10¹
2. 1 × 10¹
3. 2 × 10⁻²
4. 1 × 10⁻²
5. 1 × 10⁻³

**Question 12**

The consumption of sparkling wine in Brazil has increased in recent years. One stage of its production involves bottling the beverage in bottles similar to the one shown in the image. In this process, the liquid flow rate into the bottle remains constant and stops when it reaches the fill level.


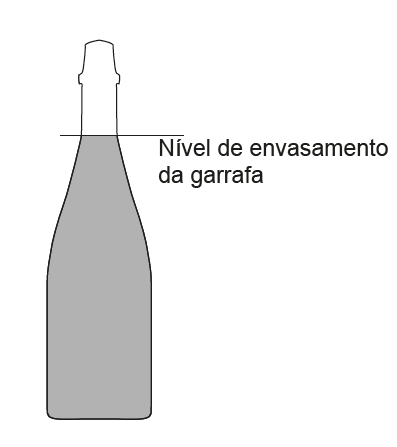


Which graph best represents the variation in the liquid height as a function of time in the bottle shown in the image?


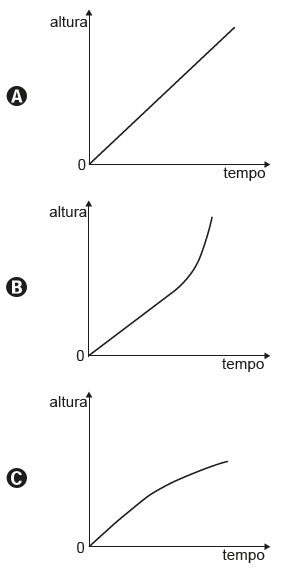

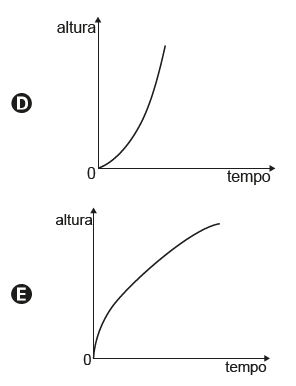


1.2) Exam B

**Question 1.**

Adam, even if we assumed that his rational faculties were entirely perfect from the beginning, could not have inferred from the fluidity and transparency of water that it would suffocate him, nor from the brightness and heat of fire that it could consume him. No object ever reveals, through the qualities that appear to the senses, either the causes that produced it or the effects that will result from it; nor is our reason capable of deriving, without the aid of experience, any conclusion concerning the actual existence of things or matters of fact.

HUME, D. An Enquiry Concerning Human Understanding. São Paulo: Unesp, 2003.

According to the author, what is the source of human knowledge?

1. The innate power of the mind.
2. The revelation of divine inspiration.
3. The study of philosophical traditions.
4. The experience of worldly phenomena.
5. The development of abstract reasoning.

**Question 2.**

To state that modern-era cartography contributed to the European invention of America means that Indigenous knowledge of the territory was ignored by European cartography, or that Indigenous peoples were deprived of their territorial representation and the authority their knowledge held over space.

OLIVEIRA, T. K. Deconstructing Maps, Revealing Spatializations: Reflections on the Use of Cartography in Studies on Colonial Brazil. Revista Brasileira de História, no. 68, 2014 (adapted).

According to the text, the cartographic representation of America was characterized by

1. the assertion of native culture.
2. advancements in environmental studies.
3. the reinforcement of forms of domination.
4. the accuracy of regional demarcations.
5. the improvement of the concept of borders.

**Question 3.**

The collision between a continental plate and an oceanic plate causes the latter to subduct beneath the continental plate, which, like island arcs, will generate a magmatic arc on the edge of the continent. This arc is composed of volcanic rocks, along with deformations and metamorphism of both pre-existing rocks and some of the newly formed rocks.

TEIXEIRA, W. et al. (Eds.). Deciphering the Earth. São Paulo: Oficina de Textos, 2000.

What physiographic feature is created by this tectonic process?

1. Abyssal plains.
2. Crystalline plateaus.
3. Absolute depressions.
4. Sedimentary basins.
5. Modern fold mountains.

**Question 4.**

A database (Table 1) presents hypothetical DNA sequences from two legally extractive areas (A1 and A2) and two conservation areas (B1 and B2). An environmental monitoring agency received an anonymous report that five furniture stores (1, 2, 3, 4, and 5) were allegedly selling products made from wood sourced from restricted extraction areas. The DNA sequences from the confiscated batches were determined (Table 2).

MIRANDA, N. E. O.; ALMEIDA JÚNIOR, E. B. A.; COLLEVATTI, R. G. Genetics Against Environmental Crimes: Identification of Illegal Timber from Conservation Units Using Molecular Markers. Genética na Escola, vol. 9, no. 2, 2014 (adapted).

**Table 1**

**
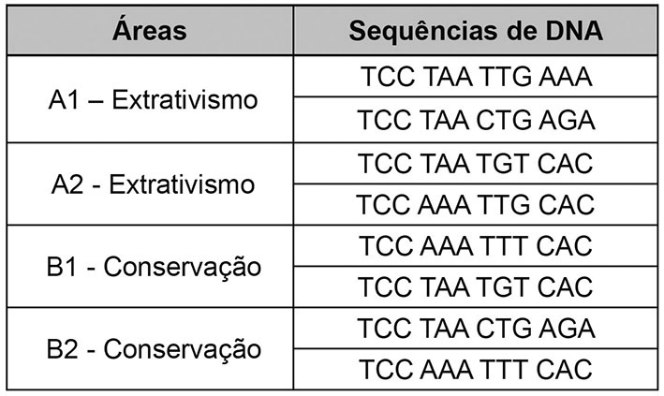
**

**Table 2**


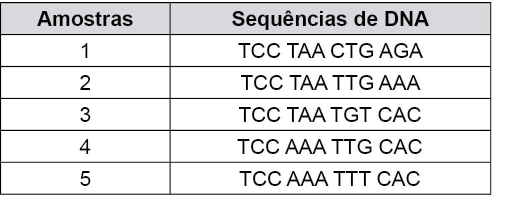


Which furniture store sells wood exclusively from illegal sources?

1. 1
2. 2
3. 3
4. 4
5. 5

**Question 5.**

Solar winds are phenomena characterized by streams of charged particles ejected from the Sun at high speed into space. Only a small fraction of these particles reaches the Earth's atmosphere at the poles, creating auroras. If these particles were to reach the surface, they could cause undesirable effects, such as interference in telecommunications, air traffic, and electrical transmission lines.

These effects are minimized on Earth by its

1. ionosphere.
2. geomagnetic field.
3. ozone layer.
4. gravitational field.
5. atmosphere.

**Question 6.**

You have been hired to synchronize the four traffic lights on an avenue, designated as O, A, B, and C, as shown in the figure.


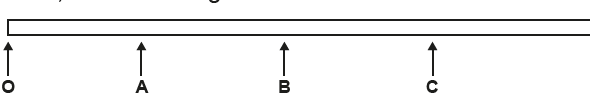


The traffic lights are spaced 500 meters apart. According to statistical data from the traffic control company, a vehicle initially stopped at light O typically accelerates at a constant rate of 1 m/s² until reaching a speed of 72 km/h, after which it continues at a constant velocity. You must adjust the timing of traffic lights A, B, and C so that they turn green when the vehicle is 100 meters away from crossing them, ensuring that it does not need to slow down at any point.

Under these conditions, approximately how long after traffic light O turns green should traffic lights A, B, and C change to green, respectively?

1. 20 s, 45 s, and 70 s.
2. 25 s, 50 s, and 75 s.
3. 28 s, 42 s, and 53 s.
4. 30 s, 55 s, and 80 s.
5. 35 s, 60 s, and 85 s.

**Question 7.**

When trying to speed up the selection process for new students, the prestigious British medical school St. George’s used a software program to determine who should be interviewed. By replicating how employees made this choice, the program automatically eliminated 60 out of 2,000 applicants—solely based on gender or racial background, inferred from their surname and place of birth. A study on this case was published in 1988, but 25 years later, another study revealed that this type of discrimination remains prevalent. A recent example involves Google’s search engine: when typing names commonly associated with Black people in the U.S., the likelihood of automatic ads suggesting background checks can increase by 25%. Worse still, searches may prompt the word “arrested?” to appear right after the queried name.

Available at: https://tab.uol.com.br. Accessed on: Aug. 11, 2017 (adapted).

The text exposes societal issues by linking information and communication technologies with

1. software efficiency.
2. the passage of time.
3. language.
4. prejudice.
5. education.

**Question 8.**

Body Slam is a gathering designed for both Deaf and hearing individuals, held since 2014 in São Paulo. It is a pioneering initiative by the group *Corposinalizante*, founded in 2008. (Before we continue, a clarification: the term slam comes from English and, in a new meaning beyond its usual sense of “hitting hard,” refers to “spoken poetry performed in rhythm with words and the city”). In these poetry slams, the initial goal was to bring Brazilian Sign Language (Libras) poetry into the scene, allowing Deaf individuals to take part and explore the intersection between poetry and sign language—essentially, a meeting of two languages. The format is simple: original poems, three minutes, one microphone. No costumes, props, or musical accompaniment. What matters is how voice and body are modulated—an artisanal process of making words “visible,” in a space where the ultimate goal is to move the audience, provoking reactions through humor, horror, chaos, sweetness, and a range of other emotions.

NOVELLI, G. Embodied Poetry. Revista Continente, no. 189, Sept. 2016 (adapted).

In the artistic practice described in the text, the body plays a central role by integrating different languages to

1. impart rhythm and visibility to poetic expression.
2. redefine the spaces where urban poetry circulates.
3. encourage original works by users of Libras.
4. translate verbal expressions into sign language.
5. provide aesthetic performances for Deaf individuals.

Question 9.

I travel through Curitiba, the city of Positivist conferences—there are eleven here, and thirteen worldwide; through the barrel organ player who hasn’t turned the crank since his little monkey died; of the brave firefighters speeding in their red truck toward a fire no one has seen, I travel through this Curitiba and the one where a hot dog comes with a double draft beer at *Buraco do Tatu*.

Curitiba, the one of the Wild Donkey, a mysterious citizen died in Rosicler’s arms—who was he?

who wasn’t he? He was the little king of Siam; of the station’s Black—the only bridge in the city, with no river beneath it, this is the Curitiba I travel through.

Curitiba, without pines or a blue sky, for which you are — province, prison, home—this Curitiba, not the one for the Englishmen to see, with love I travel through, travel through, travel through.

TREVISAN, D. In Search of the Lost Curitiba. Rio de Janeiro: Record, 1992.

The theme of Curitiba is frequent in Dalton Trevisan’s work. In this excerpt, the narrator’s relationship with the urban space is characterized by a perspective that is

1. devoid of affection, satirizing Curitiba’s customs and traditions.
2. marked by negativity, challenging conventional portrayals of the city.
3. filled with melancholy, lamenting a lack of cultural identity amid urbanization.
4. enchanted by the city’s simplicity, indifferent to its historically significant elements.
5. detached from the narrated elements, using the perspective of a traveler to express estrangement.

**Question 10.**

The table represents a family's monthly expenses (in reais) for internet, school tuition, and their child’s allowance.

| Internet | School Tuition | Child’s Allowance |
| --- | --- | --- |
| 120 | 700 | 400 |

At the beginning of the year, the costs for internet and school tuition increased by 20% and 10%, respectively. To maintain the same total monthly spending for these items, the family decides to reduce their child’s allowance.

What will be the percentage reduction in the child’s allowance?

1. 15.0%
2. 23.5%
3. 30.0%
4. 70.0%
5. 76.5%

**Question 11.**

A rectangular prism-shaped container is filled with water to a height of 8 cm. A floating object is inside.

To remove the floating object, the water level must reach at least 15 cm. To raise the water column to this height, small spheres, each with a volume of 6 cm³, will be fully submerged into the container.


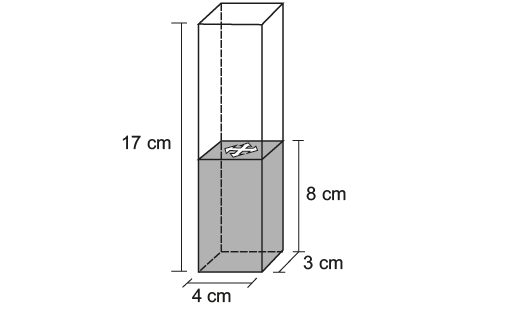


What is the minimum number of spheres required to remove the floating object, following the given instructions?

1. 14
2. 16
3. 18
4. 30
5. 34

**Question 12.**

At the nuclear power plant in Angra dos Reis, the waste produced over two decades of operations totals nearly 446 tons of spent nuclear fuel, which will remain radioactive for thousands of years. The Brazilian Institute of Environment and Renewable Natural Resources (Ibama) conditioned the approval of the Angra 3 plant’s operation—initially scheduled for 2014—on the approval of a definitive waste storage project. The National Nuclear Energy Commission (CNEN) pledged to present, by 2010, a model for storing radioactive waste for 500 years instead of thousands of years.

Época, Sept. 8, 2008 (adapted).

Assuming that the rate of fuel production remains constant and that a volume V is needed to store the 446 tons already produced, what is the approximate minimum volume a storage facility must have to accommodate 500 years of radioactive waste?

1. 25V
2. 149V
3. 1,340V
4. 11,150V
5. 14,887V

1.3) Exam C

**Question 1.**

Given the unity and activism of Black individuals, the nationalist government decided to implement reactionary and repressive measures—prohibition of the right to assembly, police surveillance and persecution, dissolution of political parties, torture, house arrest, and imprisonment of activists.

CHANAIWA, D. Southern Africa. In: MAZRUI, A.; WONDJI, C. (Eds.). General History of Africa: Africa Since 1935. Brasília: Unesco, 2010.

The actions of the South African state in the 1950s, as described, indicate that its leaders sought to

1. block violent demonstrations by the Boers.
2. comply with international legal provisions.
3. suppress active dissident organizations.
4. foster ethnic divisions within the opposition.
5. recruit native tribal leaders.

**Question 2.**

In Greece, the concept of the people included only those individuals considered citizens. Thus, it is possible to see that the concept of the people was very restrictive. Even taking this into account, the democratic system experienced by the Athenian Greeks in the 4th and 5th centuries BC can be fundamentally characterized as direct.
MANDUCO, A. Political Science. São Paulo: Saraiva, 2011.

In that context, the emergence of the government system mentioned in the excerpt promoted

1. competition for the selection of representatives.
2. a campaign to revitalize oligarchies.
3. the establishment of temporary mandates.
4. the decline of organized civil society.
5. participation in the exercise of power.

**Question 3.**

In ascetic Protestantism, we find not only a clear notion of the primacy of ethics over the world but also the mitigation of the effects of Jewish dual morality (one internal morality for fellow believers and another external one for infidels). The challenge here is ethics, which aims to cease being an occasional ideal (demanding from virtuous religious individuals an almost constant "flight from the world," as in medieval Christian monastic practice) to become effectively a practical and daily law "within the world."
SOUZA, J. The Protestant Ethic and the Ideology of Brazilian Backwardness. Brazilian Journal of Social Sciences, no. 38, Oct. 1998.

Revisiting Max Weber’s thought, the text presents the tension between ethical-religious positivity and worldly spheres of action. From this perspective, the Protestant ethic is understood as

1. linked to the abandonment of earthly happiness.
2. opposed to liberal economic principles.
3. promoting the political dimension of daily life.
4. encouraging social equality as a divine right.
5. suited to the development of modern capitalism.

**Question 4.**

To ensure the good quality of its product, a glass manufacturing company analyzed a batch of silicon dioxide (SiO2), the main component of glass. To do this, they subjected a sample of this oxide to heating until complete fusion and boiling, ultimately obtaining a temperature (T °C) versus time (t min) graph. After analyzing the graph, the analyst concluded that the sample was pure.

Data for SiO2:

Melting point = 1,600 °C; Boiling point = 2,230 °C.

Which graph was obtained by the analyst?
1)


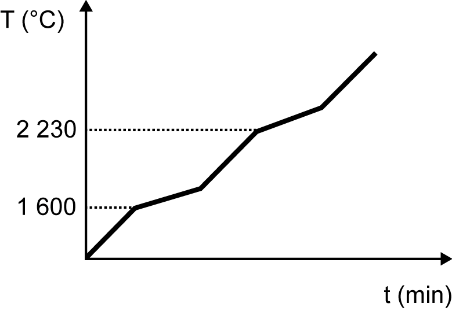


2)


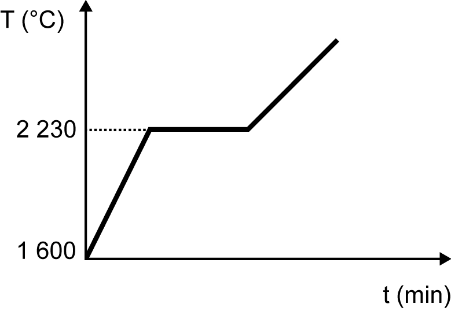


3)

**
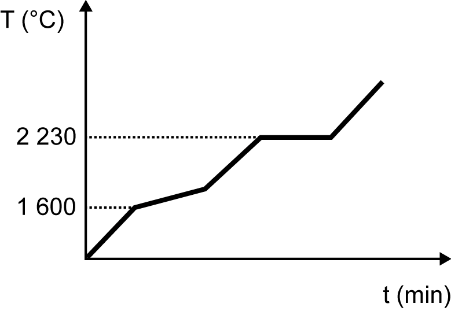
**

4)

**
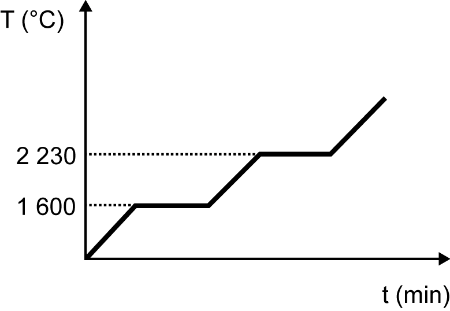
**

5)


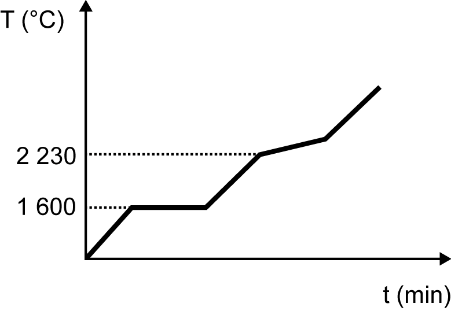


**Question 5.**

Silver objects tend to darken over time due to contact with sulfur compounds, forming a surface layer of silver sulfide (Ag2S), which is dark. A very simple method to restore the original surface of these objects is to immerse them in a heated diluted solution of sodium hydroxide (NaOH), contained in a common aluminum pan. The chemical equation illustrating this process is:

3 Ag2S(s) + 2Al(s) + 8NaOH(aq) → 6Ag(s) + 3Na2S(aq) + 2NaAlO2(aq) + 4H2O(l)

The restoration of the silver object occurs because

1. silver reduces sulfur.
2. the sulfide ion undergoes oxidation.
3. the hydroxide ion acts as an oxidizing agent.
4. aluminum acts as a reducing agent in the process.
5. the pH of the reaction medium increases during the reaction.

**Question 6**

Sucrase (or invertase) is an enzyme that acts in the human intestine by hydrolyzing the disaccharide sucrose into the monosaccharides glucose and fructose. In a kinetic study of the sucrose hydrolysis reaction (C₁₂H₂₂O₁₁), 171 g of sucrose was dissolved in 500 mL of water. It was observed that every 100 minutes of reaction, the sucrose concentration was reduced by half, regardless of the initial time chosen. The molar masses of the elements H, C, and O are 1, 12, and 16 g/mol, respectively.

What is the concentration of sucrose after 400 minutes from the start of the hydrolysis reaction?

1. 2.50 × 10⁻³ mol/L
2. 6.25 × 10⁻² mol/L
3. 1.25 × 10⁻¹ mol/L
4. 2.50 × 10⁻¹ mol/L
5. 4.27 × 10⁻¹ mol/L

**Question 7**

**Why the Stage Entrepreneurship Industry Will Destroy You**

In the past, huge books with their seven hundred pages were filled with formulas, equations, and calculations that taught you how to manage your company's cash flow. Today, they say: "You will get there! Believe it, you will win!"

*Mindset, empowerment, millennials, networking, coworking, deal, business, deadline, salesman with a hunter profile*... all of this is part of your vocabulary [translator’s note: the words in italic are in English in the original version]. The book package is always identical, and the experiences are shared in the same way: you are just one centimeter away from victory. Don’t stop!

If you give up now, it will be forever. Here, read "The Blue Ocean Strategy." Take one more mentorship session, attend another coaching session. The problem is that your mindset is not adjusted. You need to be more proactive. Let’s do another powermind? I can get you a great deal…

Source: CARVALHO, Í. C. Available at: https://medium.com. Accessed on: August 17, 2017 (adapted).

According to the text, it is possible to identify the "stage entrepreneur" by:

1. The books they recommend.
2. Their proficiency in the English language.
3. The experiences they share.
4. The language patterns they use.
5. The affordable prices of their training sessions.

**Question 8**

We went to talk to the foreman, then to an engineer, then to a supervisor, who called an engineer from our company. These men work for your company, engineer, he said, they are asking to quit. The company is committed to this bridge, folks, said the engineer, you can't just leave like that. There was a circular saw cutting some beams nearby, so we could only talk when the saw stopped, and that was getting on our nerves.

I said that we had the right to leave whenever we wanted, and that was it. Then a guy in a jacket but without a tie showed up. The engineer kept talking, and the saw kept cutting.

When he stopped talking, 50 Volts took advantage of a pause in the saw's noise and said that we were not animals to work like that.

Then the supervisor said that if it was a lack of women, they could arrange something.

The engineer said that there were more than twenty companies working on the bridge, most of them at a loss, because it was a matter of honor—we had to finish the bridge. Our company will never forget our work here on this bridge. It’s a national pride.

Source: PELLEGRINI, D. The Biggest Bridge in the World. In: Best Short Stories. São Paulo: Global, 2005.

The workers’ claims about the degrading working conditions they were subjected to receive some neutralization attempts from employer representatives, the strongest of which was:

1. A sequence of delegating responsibilities and decision-making power to third parties.
2. An appeal to financial losses and commitments related to completing the project.
3. Intimidation through the discreet presence of a security agent in the scene.
4. A promise of immediate fulfillment of the workers' sexual deprivation.
5. An appeal to identification with the company, extended to patriotic pride.

**Question 9**

**
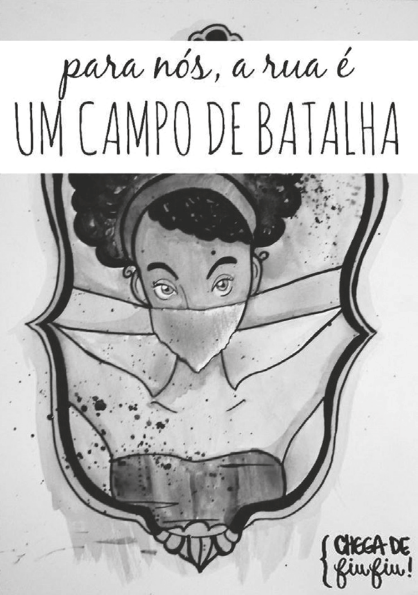
**

Translation: for us, the streets are a battle field. No more “woo hoos”.

Available at: www.bhaz.com.br. Accessed on: June 14, 2018.

This awareness campaign about the harassment suffered by women in public spaces is constructed through the combination of verbal and non-verbal language. The image of a woman with her nose and mouth covered by a scarf is a non-verbal representation of:

1. The silence imposed on women, who cannot report the harassment they suffer.
2. A metaphor suggesting that women need to defend themselves from male harassment.
3. The embarrassment women go through, leading them to try to hide themselves.
4. The necessity for women to remain unnoticed to avoid harassment.
5. The inability of women to protect themselves from verbal aggression by harassers.

**Question 10**

According to a veterinarian's recommendation, a small dog, during the first two months of life, should be fed 50 g of supplement per day and bathed four times per month. A small dog owner, following this veterinarian’s advice, purchased products/services from a certain pet shop, where the prices were presented in the following table.

| **Products/Services** | **Price** |
| --- | --- |
| Suplement | R$ 8,00 (package of 500g) |
| Bath | R$ 30,00 (unitary price) |

In the following month, the manufacturer increased the price of the supplement, which at this pet shop rose to R$ 9.00 per 500 g package. To maintain the same monthly expenses for the dog owner, the pet shop manager decided to reduce the price per bath. For calculation purposes, consider a 30-day commercial month.

Source: http://carodinheiro.blogfolha.uol.com.br. Accessed on: January 20, 2015 (adapted).

Under these conditions, the new unit price of the bath (in Brazilian reais) became:

1. 27.00
2. 29.00
3. 29.25
4. 29.50
5. 29.75

**Question 11**

Consider the crane shown in the figures, in two positions (1 and 2). In position 1, the boom forms a right angle with the steel cable (CB) that holds a metal sphere at its lower end. In position 2, the crane raised its boom, and the new angle between the boom and the steel cable (ED), which holds the metal ball, is now 60°.


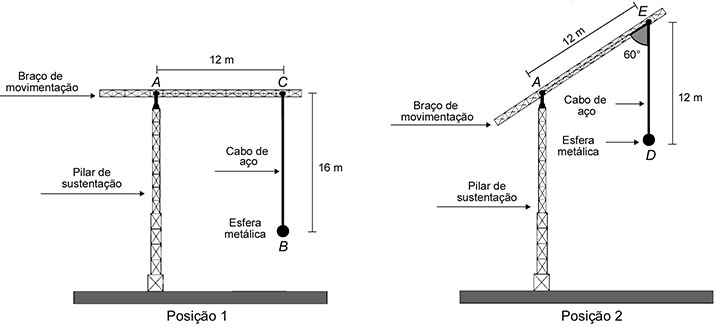


Given that points A, B, and C in position 1 form triangle T1 and points A, D, and E in position 2 form triangle T2, they can be classified as obtuse, right, or acute and as equilateral, isosceles, or scalene.

According to these classifications, triangles T1 and T2 are identified as:

1. Right scalene and right isosceles.
2. Acute scalene and right isosceles.
3. Right scalene and acute scalene.
4. Acute scalene and acute equilateral.
5. Right scalene and acute equilateral.

**Question 12**

A chocolate company consulted its production manager and verified that five different types of chocolate bars could be produced, with the following market prices:

- Bar I: R$ 2.00
- Bar II: R$ 3.50
- Bar III: R$ 4.00
- Bar IV: R$ 7.00
- Bar V: R$ 8.00

Analyzing market trends, the sales manager determined that profit (L) is given by the function: L(x) = –x² + 14x – 45, where x is the price of the chocolate bar.

The company should invest in producing the chocolate bar that yields the highest profit. Which chocolate bar should the company invest in producing?

1. I
2. II
3. III
4. IV
5. V
